# Natural killer cell dysfunction in glioma: from immune evasion to immunotherapy

**DOI:** 10.3389/fimmu.2026.1787023

**Published:** 2026-03-18

**Authors:** Run Zhang, Pengcheng Ma, Ke Tang, Yanchun Cao, Yani Yang, Shengyang Hao, Tingting Li, Xiaoming Peng

**Affiliations:** 1 School of Traditional Chinese and Western Medicine, Gansu University of Chinese Medicine, Lanzhou, Gansu, China; 2 Department of Neurology, Affiliated Hospital of Gansu University of Chinese Medicine, Lanzhou, Gansu, China; 3 Department of Psychiatry, Lanzhou Second People’s Hospital, Lanzhou, Gansu, China

**Keywords:** CAR-NK therapy, glioblastoma, immune checkpoint inhibition, immune evasion, immunosuppression, natural killer cell, tumormicroenvironment

## Abstract

Natural killer (NK) cells, critical components of innate immunity, possess the ability to eliminate tumor cells without prior sensitization. In gliomas, particularly glioblastoma, the tumor microenvironment (TME) exerts potent immunosuppressive effects that impair NK cell function through MHC-I overexpression, secretion of TGF-β and IDO, and recruitment of myeloid-derived suppressor cells (MDSCs). Emerging evidence highlights the significance of NK cell infiltration, cytotoxicity, and ligand-receptor dynamics—such as NKG2D, KIRs, and CX3CR1^+^ subsets—in shaping prognosis and therapeutic responsiveness in glioma patients. Therapeutic strategies including activation of NK cells via chemotherapeutics (bortezomib, decitabine), blockade of inhibitory receptors (NKG2A, CD161), and combinatorial approaches with immune checkpoint inhibitors are under active investigation. Notably, chimeric antigen receptor (CAR)-engineered NK cells targeting EGFR, HER2, GD2, and CD133 show promise in preclinical glioma models due to their enhanced specificity and reduced toxicity compared to CAR-T cells. This review summarizes the multifaceted roles of NK cells in glioma immunity and highlights novel immunotherapeutic strategies to restore NK cell function and improve clinical outcomes.

## 1 Introduction

Gliomas, particularly glioblastoma (GBM), constitute the most aggressive and fatal category of primary brain malignancies, distinguished by diffuse parenchymal infiltration, rapid proliferative capacity, and marked resistance to conventional therapies (1, 2). Gliomas are sustained by a profoundly immunosuppressive tumor microenvironment (TME) that constrains effective antitumor immunity (3, 4). Natural killer (NK) cells, as innate immune effectors capable of eliminating transformed cells without prior antigen sensitization, hold substantial promise in GBM (5). However, glioma progression is frequently associated with diminished NK cell infiltration and dysfunction driven by TME-mediated inhibitory signals (6, 7).

Recent studies have underscored the bidirectional crosstalk between NK cells and glioma cells as a determinant of disease trajectory and therapeutic responsiveness (7). NK cell activity is governed by a finely tuned equilibrium between activating receptors, such as NKG2D and NKp30, and inhibitory pathways mediated by major histocompatibility complex class I (MHC-I) engagement and soluble suppressive factors including TGF-β and indoleamine 2,3-dioxygenase (IDO), which are frequently dysregulated in gliomas (5, 8). Furthermore, the emergence of engineered immunotherapies, such as CAR-NK cells targeting glioma-specific antigens (EGFR, HER2, CD133), has broadened the therapeutic landscape (6). This review summarizes current knowledge of NK cell biology in the glioma microenvironment, delineates the molecular and cellular mechanisms underpinning NK cell suppression, and critically evaluates emerging therapeutic strategies designed to restore NK cell effector function in glioma immunotherapy.

## 2 Phenotypic subsets and receptor-mediated regulation of NK cell cytotoxicity

NK cells comprise two principal subsets defined by differential CD56 and CD16 expression (7). The CD56^bright^CD16^-^ NK cells, enriched in secondary lymphoid tissues, exert immunoregulatory functions through the secretion of interferon-γ (IFN-γ), tumor necrosis factor-α (TNF-α), and granulocyte–macrophage colony-stimulating factor (GM-CSF), thereby promoting tumor cell apoptosis and growth restraint (8, 9). In contrast, CD56 ^dim^CD16^+^ NK cells predominate in peripheral blood and mediate direct cytotoxicity via perforin and granzymes, while also executing antibody-dependent cellular cytotoxicity (ADCC) through CD16 engagement of antibody-coated targets (9). Through their capacity to eliminate infected or transformed cells without prior sensitization, NK cells constitute a rapid and essential arm of innate immune surveillance against viral infection and oncogenesis (10–12). NK cell effector function is governed by the integration of activating (activatory killer receptors, AKRs) and inhibitory (inhibitory killer receptors, IKRs) signals (13, 14). Activating receptors include natural killer cell receptor protein 1 (NKR-P1), natural cytotoxicity receptors (NCRs), and natural killer group 2D (NKG2D) (15, 16). In parallel, inhibitory receptors—some functioning independently of MHC-I—such as T cell immunoglobulin and mucin domain-3 (TIM-3), T cell immunoglobulin and ITIM domains (TIGIT), poliovirus receptor-related immunoglobulin domain-containing protein (PVRIG), and programmed death-1 (PD-1) (17–19). The balance of these opposing signals enables NK cells to detect and eliminate targets in an MHC-I–independent manner, a property particularly critical in tumor immunity, where malignant cells frequently downregulate MHC-I to evade cytotoxic T lymphocyte recognition (20). Consequently, NCR- and NKG2D-dependent surveillance by NK cells represents a pivotal mechanism counteracting tumor immune escape.

## 3 NK cell interactions with glioma microenvironment

### 3.1 NK cell exhaustion in the gliomas

Gliomas are widely regarded as immunologically ‘cold’ tumors, characterized by sparse lymphocyte infiltration and limited responsiveness to conventional immunotherapies (21, 22). TME exerts profound systemic and local immunosuppressive effects, constraining innate and adaptive immunity. NK cells, although variably abundant, show clinical correlations with patient outcomes (23, 24). Under physiological conditions, NK cytotoxicity is tightly calibrated by inhibitory receptors engaging MHC-I on healthy cells. In gliomas, however, tumor cells evade NK-mediated clearance through enhanced cytokine secretion and upregulated MHC-I expression (25). Ligation of killer immunoglobulin-like receptors (KIRs) initiates immunoreceptor tyrosine-based signaling cascades that ordinarily culminate in tumor cell lysis (26). Paradoxically, high MHC-I expression in most gliomas reinforces KIR-mediated inhibitory signaling, facilitating immune escape (27). NK cells express NKG2D, an activating receptor that does not depend on MHC-I; thus, the abundance of NKG22D ligands within the tumor microenvironment critically influences NK cell function (28, 29). In gliomas, tumor cells are capable of altering the expression profiles of surface receptors on NK cells, resulting in functional suppression (30). Frequent IDH1/2 mutations drive epigenetic reprogramming of immune-related genes, suppress NKG2D ligand expression, and thereby enhance resistance to NK cytotoxicity (31, 32). Notably, IDH1-R132H–mutant tumors can reshape immune infiltration patterns and associate with improved prognosis, underscoring context-dependent NK–glioma crosstalk (33, 34).

### 3.2 Cytokine-mediated suppression of NK cell function

In addition to modulating the expression of activating ligands such as NKG2D, gliomas actively subvert natural killer (NK) cell immunity through the secretion of immunosuppressive cytokines that reprogram the tumor microenvironment (6, 35). Transforming growth factor-β (TGF-β) emerges as a central regulator of NK cell dysfunction within glioblastoma (36, 37). Glioma stem cells (GSCs), a critical driver of tumor propagation and therapeutic resistance, constitutively produce TGF-β, thereby reshaping the immune landscape toward suppression (38, 39). Engagement of αv integrins present on GSCs with CD9 and CD103 molecules located on NK cells stimulates TGF-β secretion by GSCs. The released TGF-β subsequently signals through transforming growth factor beta receptor 2 (TGFBR2) on NK cells, activating downstream pathways that attenuate cytotoxic effector functions and cytokine production (36, 40). The anti-tumor activity of NK cells can be restored when donor-derived or allogeneic NK cells are administered in combination with pharmacological blockade of αv integrins or TGF-β receptors (36, 41). Beyond local tumor–NK interactions, TGF-β exerts systemic immunomodulatory effects. In peripheral blood NK cells, TGF-β reduces the expression of the activating receptor NKG2D, thereby dampening target-cell recognition and cytolytic competence (42, 43). These findings position TGF-β not merely as a soluble suppressive factor but as a master regulator of NK cell phenotypic and functional plasticity in glioma.

**Figure 1:**
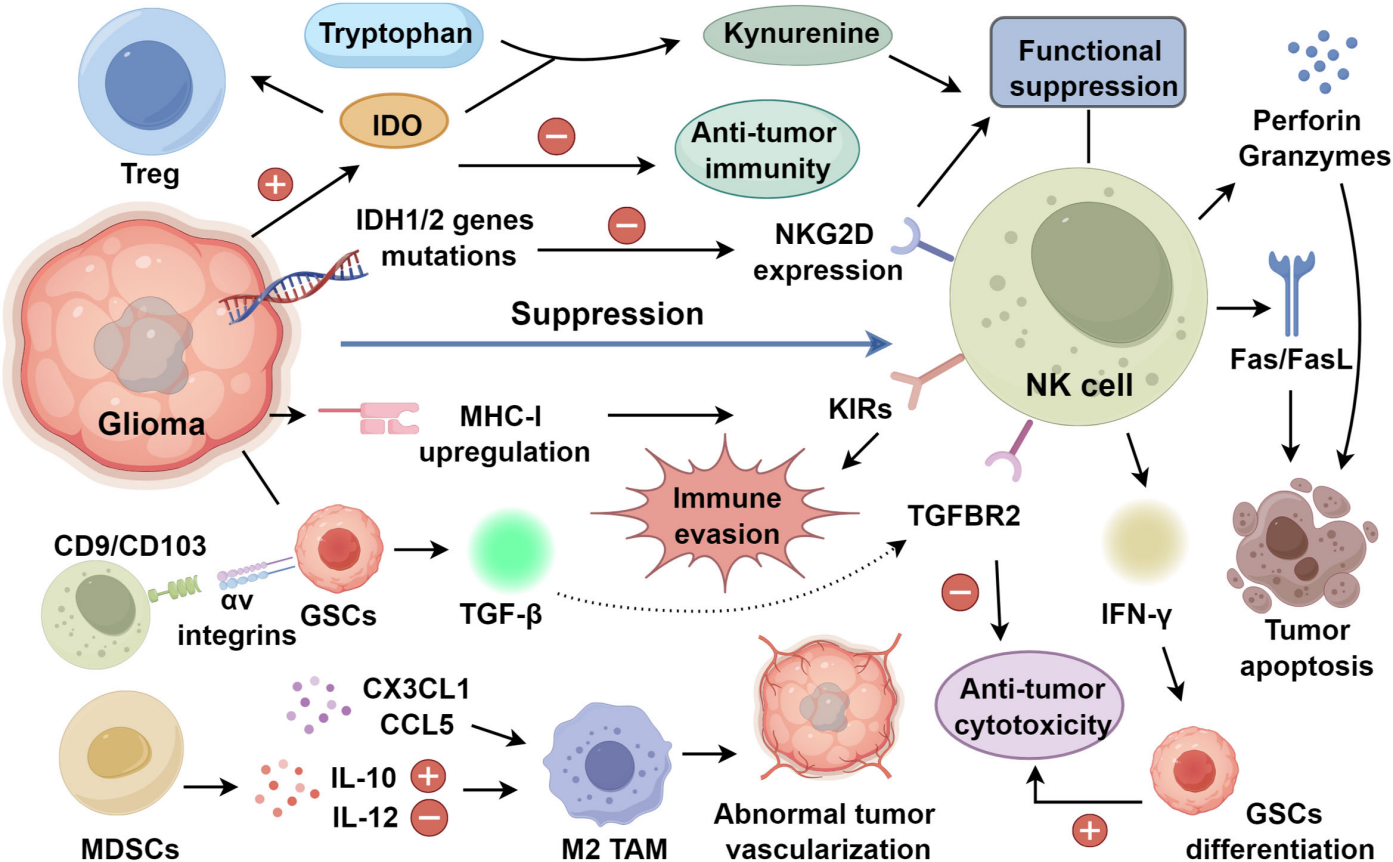
NK cell suppression in glioma progression.

Metabolic stress further compounds cytokine-mediated immune suppression. Rapid glioma proliferation generates a hypoxic and nutrient-deprived microenvironment that constrains NK cell bioenergetics and effector capacity (44, 45). In particular, lactate accumulation and tryptophan depletion represent key metabolic perturbations that impair NK cell activation and proliferative responses (46, 47). A pivotal mediator of this metabolic immune checkpoint is IDO, whose expression is frequently upregulated in gliomas and correlates with suppressed anti-tumor immunity (8, 48). As the principal rate-limiting enzyme of tryptophan catabolism, IDO catalyzes the conversion of tryptophan into kynurenine. Elevated kynurenine levels induce NK cell dysfunction while concurrently promoting the expansion of immunosuppressive regulatory T cells (Tregs), thereby orchestrating a dual mechanism of immune evasion (49, 50) (**Figure 1**).

### 3.3 MDSC and NK cell network in gliomas

Patients with gliomas exhibit a marked expansion of myeloid-derived suppressor cells (MDSCs) in peripheral blood compared with healthy individuals (51), underscoring their systemic immunomodulatory influence. MDSCs potentiate interleukin-10 (IL-10) production through reciprocal interactions with macrophages while attenuating interleukin-12 (IL-12) secretion, thereby skewing macrophage polarization toward a M2 phenotype (52, 53). Concomitantly, chemokine axes (CX3CL1, CCL5) facilitate the recruitment and retention of glioma-associated macrophages, reinforcing aberrant vascularization and sustaining a permissive niche for tumor progression (54, 55). Metabolic competition within the hypoxic and nutrient-depleted glioma milieu further compromises immune effector function. Tumor-driven sequestration of oxygen and key metabolites perturbs the bioenergetic and metabolic circuitry of NK cells, attenuating their cytotoxic competence (56, 57). Additionally, malignant cells evade NK cell–mediated surveillance by downregulating tumor-associated antigen expression and inhibiting processes such as trogocytosis, thereby diminishing effective target recognition and lytic engagement (20, 58). In gliomas, this MDSC–NK cell axis orchestrates immune evasion and reveals actionable vulnerabilities. Therapeutic strategies aimed at disrupting chemokine-mediated myeloid recruitment, restoring metabolic fitness, or reactivating NK cell receptor–ligand signaling may reinvigorate NK cell–driven immunosurveillance and improve clinical outcomes in glioma (59).

### 3.4 Clinical advances and translational challenges of NK-based glioma immunotherapies

In gliomas, NK cells exhibit dynamic alterations in abundance and functional competence, with their immunological fitness closely mirroring clinical trajectory. Neuron-derived CX3CL1 orchestrates the recruitment of CX3CR1^+^ NK cells into the central nervous system, establishing a chemokine axis that underpins immune surveillance (7, 60). When compared to lower-grade gliomas (LGG), glioblastoma tissues show a pronounced decrease in NK cell infiltration alongside a functional impairment, which fosters tumor advancement and recurrence (61). Single-cell transcriptomic profiling further delineates a KIR2DS2^+^ NK cell subset endowed with potent cytotoxic capacity through engagement of tumor-expressed NKG2D ligands; notably, the abundance of these effector cells declines in parallel with increasing tumor grade (62, 63). Intriguingly, NK cell infiltration in GBM surpasses that observed in breast cancer or melanoma, underscoring their non-redundant role in glioma immune surveillance (64). NK cell presence has also been documented in meningiomas and cerebral metastases, and *in vitro* studies confirm efficient NK cell–mediated lysis of GBM and medulloblastoma cells (60, 65).

Clinical investigations corroborate systemic perturbations in NK cell subsets across glioma grades. Patients with high-grade gliomas (HGG) display a marked reduction in circulating CD56^bright^ NK cells relative to those with LGG, a deficit associated with unfavorable outcomes (66). A phase I/IIa trial demonstrated that adoptive NK cell immunotherapy significantly prolonged overall and progression-free survival in recurrent glioma, highlighting translational feasibility (67). These findings position NK cell abundance and functional integrity as determinants of prognosis and therapeutic responsiveness, supporting strategies aimed at restoring NK cell infiltration and effector capacity within the tumor microenvironment. NK cells display strong cytotoxic activity targeting glioma stem cells (GSCs). Following tumor cell recognition, direct cytolysis can be initiated by NK cells through the release of perforin and granzymes or through pathways reliant on death receptors (68). Beyond cytotoxic elimination, NK cell–derived IFN-γ promotes GSC differentiation, thereby enhancing chemosensitivity and diminishing resistance to NK-mediated killing (69). These dual cytolytic and differentiation-inducing functions underscore the therapeutic rationale for augmenting NK cell activity against GSCs and harnessing IFN-γ–driven reprogramming as an integrated strategy for glioma control (7, 70).

## 4 NK cell-based immunotherapeutic strategies for gliomas

### 4.1 NK cells activation in glioma therapy

NK cell activity is tightly regulated by a dynamic balance between activating and inhibitory receptor signaling, which determines their capacity for immune surveillance and cytotoxicity. Beyond their canonical lytic function, accumulating evidence indicates that selected chemotherapeutic and molecularly targeted agents can functionally reprogram NK cells (71, 72). These effects are mediated through upregulation of activating receptors, enhanced cytokine production, and increased expression of stress-induced ligands for the activating receptor NKG2D on tumor cells. Notably, activation of the DNA damage repair (DDR) pathway has emerged as a critical upstream signal driving the induction of NKG2D ligands, thereby amplifying NK cell–mediated antitumor responses (73, 74). For instance, the proteasome inhibitor bortezomib (BTZ) activates DDR signaling and promotes the expression of ligands for NKG2D and DNAM-1, resulting in augmented NK cell cytotoxicity against glioblastoma cells. Preclinical studies further demonstrate that the combined administration of autologous NK cells and BTZ significantly delays tumor progression and prolongs survival (75, 76). Similarly, the hypomethylating agent decitabine re-sensitizes IDH-mutant glioma cells to NK cell–mediated lysis through epigenetic upregulation of NKG2D ligands (31, 77). Furthermore, mocetinostat, an inhibitor of histone deacetylase, enhances the expression of NKG2D ligands and increases the susceptibility of GBM cells to NK cell-mediated killing (78). In recent studies, trifunctional antibodies that bind tumor antigens while concurrently targeting NKp46 and CD16 on NK cells have exhibited enhanced antitumor activity relative to standard tumor-directed antibodies in both experimental and animal models (79, 80). MICA and MICB, encoded by the MHC class I–related genes A and B, serve as endogenous ligands for the activating receptor NKG2D. Tumor-associated proteolytic shedding of MICA/MICB impairs immune surveillance by attenuating NKG2D-dependent recognition (81, 82). Therapeutic approaches that prevent this proteolytic release preserve ligand density at the tumor cell surface, thereby sustaining NKG2D signaling and constraining tumor progression, largely through cooperative engagement of NKG2D and CD16 (83). Besides, inhibitory killer cell immunoglobulin-like receptors (KIRs) recognize MHC class I molecules expressed on tumor cells, delivering suppressive signals that dampen NK cell activation and facilitate immune evasion (84, 85).

### 4.2 NK cells based combination therapy

Combined blockade of PD-1 and CTLA-4 potentiates the recruitment of NK cells and CD8^+^ T cells into the central nervous system (CNS), resulting in markedly prolonged survival in glioblastoma mouse models (86, 87). Nevertheless, the blood–brain barrier (BBB) remains a formidable obstacle, functioning as a selectively permeable interface that restricts the entry of both immune effectors and therapeutic agents into brain parenchyma (88). To overcome this constraint, covalent conjugation of poly (β-L-malic acid) to antibodies targeting PD-1 or CTLA-4 has been shown to facilitate trans-BBB delivery, leading to enhanced NK cell accumulation and improved survival in mice bearing intracranial GL261 gliomas (89). These findings suggest that integrating strategies that promote NK cell trafficking with the intrinsic cytolytic capacity of NK cells may simultaneously counteract BBB exclusion and the profound immunosuppression characteristic of CNS tumors (7). Malignant gliomas further evade immune surveillance through upregulation of inhibitory ligands, including human leukocyte antigen E (HLA-E) and lectin-like transcript 1 (LLT1), which engage CD94/NKG2A and CD161 on NK cells to attenuate cytotoxic function (60, 90, 91). Targeted disruption of these axes via small interfering RNA or blocking antibodies restores NK cell–mediated lysis of glioma cells (60). Notably, in head and neck squamous cell carcinoma models, the humanized anti-NKG2A antibody monalizumab synergizes with the anti-epidermal growth factor receptor (EGFR) antibody cetuximab to augment antibody-dependent cellular cytotoxicity (ADCC) (92, 93). Given that EGFR constitutes a major therapeutic target in gliomas, NKG2A blockade with monalizumab may similarly enhance NK cell–mediated ADCC in this context, an approach of particular relevance for tumors refractory to temozolomide (92, 94).

## 5 Genetically engineered CAR-NK cell therapy for glioma

### 5.1 CAR-NK therapy

Chimeric antigen receptors (CARs) are synthetic immune receptors generated by replacing the variable domains of the TCR α and β chains with a tumor-specific single-chain variable fragment (scFv) derived from an antibody (95). The scFv is fused via a transmembrane domain to intracellular signaling modules, most commonly CD3ζ, thereby conferring antigen-triggered activation upon expression in T lymphocytes or NK cells to generate CAR-T or CAR-NK cells, respectively (96, 97). Through antibody-like recognition independent of major histocompatibility complex restriction, the scFv endows effector cells with precise tumor targeting and potent cytotoxicity (98). Despite their robust *in vivo* expansion and persistence, CAR-T cells are frequently constrained by severe toxicities, including cytokine release syndrome (CRS) and graft-versus-host disease (GVHD), which may be life-threatening (99, 100). In contrast, CAR-NK platforms exhibit a comparatively favorable safety profile, largely circumventing these complications while retaining intrinsic cytotoxicity. CAR-NK cells can be engineered to target diverse tumor-associated antigens, such as EGFR and human epidermal growth factor receptor 2 (HER2) (101, 102). Preclinical studies in both *in vitro* and *in vivo* glioblastoma models consistently demonstrate that CAR-NK cells elicit substantial antitumor activity (102). Therapeutic efficacy, however, is critically dependent on rational antigen selection. Ideal targets display high, homogeneous expression within tumor tissues and minimal distribution in normal organs, thereby maximizing on-tumor potency while mitigating off-tumor toxicity (103, 104).

### 5.2 Selection of targets for CAR-NK therapy

In glioblastoma, amplification and overexpression of EGFR are frequently observed, in stark contrast to its minimal expression in normal brain tissue (105). A constitutively active mutant, EGFRvIII, is commonly co-expressed in GBM and is strongly associated with adverse prognosis and diminished overall survival (106, 107). These molecular features have positioned EGFR and EGFRvIII as compelling immunotherapeutic targets. Accordingly, multiple CARs constructs recognizing distinct EGFR epitopes have been engineered (108). CAR-engineered NK-92 cells demonstrate robust cytotoxicity against xenografted gliomas overexpressing EGFR or EGFRvIII (101, 109). Studies using immunocompromised NSG mice with intracranial EGFR- or EGFRvIII-positive GBM xenografts demonstrated that repeated intracranial administration of CAR-NK-92 cells substantially curbed tumor growth and extended overall survival relative to treatment with unmodified NK-92 cells alone (101). Therapeutic efficacy can be further potentiated through combinatorial strategies. Ma and colleagues demonstrated that co-administration of an oncolytic virus encoding an IL-15/IL-15Rα fusion protein with EGFR-directed CAR-NK cells substantially enhanced antitumor activity (110). This approach achieved superior tumor control and promoted increased intratumoral NK-cell infiltration relative to CAR-NK monotherapy, underscoring the importance of cytokine support in sustaining NK-cell persistence and function within the immunosuppressive GBM microenvironment (111). In glioblastoma, HER2 overexpression has been reported in up to 70% of cases and correlates with unfavorable clinical outcomes (112). Preclinical xenograft models have demonstrated that localized administration of HER2-targeted CAR-NK cells significantly restrains tumor progression and prolongs survival (113). Notably, HER2-directed CAR-NK cells effectively eliminated HER2-positive glioblastoma stem cells (GSCs), highlighting their capacity to target tumor-initiating populations. However, prolonged coculture resulted in attenuated cytotoxic activity, suggesting adaptive resistance mechanisms and antigenic modulation may limit sustained therapeutic efficacy (114).

## 6 Conclusion

Natural killer (NK) cell dysfunction represents a central axis of immune escape in glioma, particularly in glioblastoma, where the tumor microenvironment orchestrates multilayered suppression through MHC-I upregulation, TGF-β and IDO signaling, metabolic stress, and the expansion of MDSCs. These convergent mechanisms attenuate NK cell infiltration, receptor-mediated activation, and cytotoxic competence, thereby undermining innate immune surveillance. Emerging single-cell and functional analyses underscore the heterogeneity of NK cell subsets and reveal context-dependent interactions with glioma stem cells, chemokine networks, and immune checkpoints. Collectively, these findings reposition NK cells not as passive bystanders but as dynamic regulators of tumor progression and therapeutic responsiveness.

Therapeutically, strategies aimed at restoring NK cell activity, including pharmacologic upregulation of activating ligands, blockade of inhibitory receptors, combinatorial immune checkpoint modulation, and genetically engineered CAR-NK platforms targeting EGFR, HER2, GD2, and CD133, have demonstrated promising preclinical and early clinical efficacy. Compared with CAR-T approaches, CAR-NK therapies offer a favorable safety profile and inherent antitumor versatility. Nonetheless, durable responses will likely require rational integration of cytokine support, microenvironmental reprogramming, and optimized trafficking across the blood–brain barrier. Future translational efforts should prioritize mechanistic stratification of NK cell states and precision targeting within the glioma niche, thereby advancing NK-based immunotherapy from experimental promise toward clinical reality.
